# ATP and Liv-52 Ameliorate Linezolid-Induced Liver Injury via Modulation of NF-κB/NLRP3 Pathways

**DOI:** 10.3390/biomedicines14061286

**Published:** 2026-06-04

**Authors:** Serkan Cerrah, Ahmed Ramiz Baykan, Esra Tuba Sezgin, Gulbaniz Huseynova, Elif Karabacak, Serdar Tanas, Emine Kartal Baykan, Murat Gunay, Ali Gungor, Halis Suleyman

**Affiliations:** 1 Department of Gastroenterology, Erzurum City Hospital, Erzurum 25070, Turkey; ahmedbaykan@hotmail.com (A.R.B.); serdartanas@gmail.com (S.T.); 2 Anesthesia Program, Vocational School of Health Services, Erzincan Binali Yıldırım University, Erzincan 24100, Turkey; esra.demir@erzincan.edu.tr; 3 Department of Pharmacology, Azerbaijan Medical University, Baku AZ1022, Azerbaijan; huseynovagulbaniz04@gmail.com; 4 Department of Pharmacology, Faculty of Pharmacy, Duzce University, Duzce 81620, Turkey; elifkarabacak2010@hotmail.com; 5 Department of Endocrinology and Metabolic Diseases, Erzurum City Hospital, Erzurum 25070, Turkey; emnkrtl@hotmail.com; 6 Department of Biochemistry, Erzincan Mengucek Gazi Training and Research Hospital, Erzincan 24100, Turkey; mgunay@erzincan.edu.tr; 7 Laboratory and Veterinary Health Program, Vocational School of Health Services, Osmaniye Korkut Ata University, Osmaniye 80000, Turkey; aligungor@osmaniye.edu.tr; 8 Department of Pharmacology, Faculty of Medicine, Erzincan Binali Yıldırım University, Erzincan 24100, Turkey

**Keywords:** linezolid, oxidative stress, ATP, Liv-52, NLRP3, necroptosis

## Abstract

**Objective**: Linezolid (LZD), an oxazolidinone antibiotic widely used against Gram-positive infections, has been associated with mitochondrial dysfunction and hepatotoxicity, particularly during prolonged use. This study aimed to investigate the protective effects of adenosine triphosphate (ATP) and Liv-52 against LZD-induced liver injury, with a focus on oxidative stress, inflammation, and necroptosis pathways. **Methods**: Twenty-four male Wistar rats were randomly assigned to four groups: healthy control (HG), LZD-treated (LZDG), Liv-52 + LZD (LVLZ), and ATP + LZD (ATLZ). Liv-52 (50 mg/kg, orally) and ATP (5 mg/kg, intraperitoneally) were administered prior to LZD (125 mg/kg, orally) for 14 days. **Results**: Following LZD administration, malondialdehyde (MDA) levels markedly increased, indicating oxidative stress, while total glutathione (tGSH), superoxide dismutase (SOD), and catalase (CAT) activities significantly decreased. Histopathological examination revealed pronounced hepatocellular damage accompanied by increased NF-κB, NLRP3, RIPK3, and MLKL expression, indicating activation of inflammatory and necroptotic pathways. Treatment with ATP and Liv-52 significantly ameliorated these biochemical, histopathological, and molecular alterations. **Conclusions**: Treatment with ATP and Liv-52 significantly attenuated oxidative stress, improved histopathological alterations, and suppressed the expression of inflammatory and necroptotic markers. Notably, ATP exhibited a more pronounced protective effect compared to Liv-52. In conclusion, LZD induces hepatotoxicity through oxidative stress-mediated inflammatory and necroptotic mechanisms, while ATP and Liv-52 confer hepatoprotection, with ATP showing superior efficacy.

## 1. Introduction

LZD is an oxazolidinone antibiotic used against Gram-positive bacteria, exerting its effect by inhibiting protein synthesis. Although short-term use is generally well tolerated, mitochondrial dysfunction may occur with prolonged administration [1]. Long-term LZD therapy has been associated with adverse effects such as drug-induced liver injury, lactic acidosis, peripheral neuropathy, and bone marrow suppression [2,3]. LZD directly affects mitochondria by binding to mitochondrial ribosomes; impairment of mitochondrial function shifts energy production toward anaerobic pathways, leading to lactate accumulation in the blood [4]. Mitochondrial dysfunction plays a central role in drug-induced liver injury. Impairment of oxidative phosphorylation, decreased ATP levels, and increased production of reactive oxygen species (ROS) are the main mechanisms underlying hepatocellular damage [5]. Excessive ROS generation disrupts cellular functions, leading to oxidative stress and inflammation [6]. Therefore, antioxidant compounds are frequently utilized to mitigate such damage [7,8]. Current literature indicates that oxidative stress and inflammation are key contributors to linezolid-induced toxicity. However, the molecular mechanisms underlying LZD-induced oxidative stress have not yet been fully elucidated. In light of these findings, LZD has been considered a suitable experimental model for investigating hepatotoxicity associated with oxidative stress, inflammation, and mitochondrial dysfunction. Therefore, elucidating the molecular pathways involved in LZD-induced liver injury may contribute to the development of effective hepatoprotective strategies. Additionally, previous studies have reported that linezolid reduces intracellular ATP concentrations [9]. ATP, which plays a central role in cellular energy metabolism, consists of adenine, ribose sugar, and three phosphate groups [10]. Previous studies have demonstrated that ATP deficiency may increase cellular necrosis [11]. Approaches that support cellular energy metabolism have been reported to be beneficial in the treatment of liver injury [12]. Furthermore, the antioxidant properties of ATP suggest that it may play a protective role in reducing drug-induced oxidative stress in hepatocytes [13]. Liv-52 is an Ayurvedic formulation composed of seven different plants and one mineral, including Capparis spinosa, Solanum nigrum, Cichorium intybus, Terminalia arjuna, Achillea millefolium, Tamarix gallica, Cassia occidentalis, and Mandur Bhasma [14]. Liv-52 has been shown to possess strong antioxidant, anti-inflammatory, and immunomodulatory properties [15,16]. Its protective mechanism involves preventing glutathione (GSH) depletion and reducing lipid peroxidation (LPO) [17]. These findings suggest that Liv-52 may be beneficial in modulating drug-induced oxidative stress in the liver. Previous experimental studies have also demonstrated the hepatoprotective potential of Liv-52 in various models of drug-induced liver injury. Collectively, these findings indicate that both ATP and Liv-52 may have protective potential against LZD-induced oxidative liver injury. However, no previous study has investigated the protective effects of ATP and Liv-52 on linezolid-induced hepatotoxicity. To the best of our knowledge, this is the first study to comparatively evaluate the protective effects of ATP and Liv-52 against linezolid-induced hepatotoxicity with a particular focus on NF-κB/NLRP3-mediated inflammation and RIPK3/MLKL-associated necroptosis. Evaluation of these signaling pathways may provide important mechanistic insights into the inflammatory and necroptotic processes involved in LZD-induced hepatotoxicity. Therefore, the aim of this study was to investigate and comparatively evaluate the protective effects of Liv-52 and ATP against linezolid-induced liver toxicity in rats.

## 2. Materials and Methods

### 2.1. Animals and Ethical Approval

A total of 24 male albino Wistar rats, weighing between 280 and 292 g, were included in the study. All animals were obtained from the Experimental Animals Application and Research Center of Erzincan Binali Yıldırım University (Erzincan, Türkiye). The animals were randomly divided into four groups in such a way that the mean body weights of each group were similar. Prior to the experiment, the rats were housed in groups to allow adaptation to the environment under controlled laboratory conditions, including a 12-h light/dark cycle, a temperature of 22 °C, and a humidity range of 30–70%. The animals were kept in wire cages (dimensions: length 550 mm; width 350 mm; height 200 mm; floor area 1925 cm^2^). Rats were provided with unlimited (ad libitum) access to tap water and standard pellet feed (Experimental Animal Feed; Bayramoğlu Inc., Erzurum, Türkiye). All experimental procedures were conducted in accordance with the European Parliament and Council Directive 2010/63/EU (approval number: 2016-24-199) and the ARRIVE guidelines [18]. All procedures were approved by the Local Ethics Committee for Animal Experiments of Erzincan Binali Yıldırım University (Meeting Date: 26 February 2026; Meeting No: 2026/02).

### 2.2. Chemicals

The chemicals used in the experiment were thiopental sodium (IE Ulagay, Istanbul, Türkiye), Liv-52 (Himalaya Wellness Company, Bengaluru, India), ATP (Zdorove Narodu, Kharkiv, Ukraine) and linezolid (Zyvoxid tablet; Pfizer, Istanbul, Türkiye).

### 2.3. Experimental Design

For sample size determination, the minimum number of animals required was used in accordance with the 3R principles (Replacement, Reduction, and Refinement) [19]. Animals showing signs such as hunched posture, reduced movement, or injury caused by cage mates were predefined as exclusion criteria for both the experimental process and subsequent data analysis; however, no exclusions occurred during the study. Randomization was performed using a random numbers table. Cages and animals were systematically numbered to minimize potential confounding factors.

### 2.4. Experimental Groups

The rats to be used in the experiment were divided into four groups: healthy control (HG), LZD-treated (LZDG), Liv-52 + LZD (LVLZ), and ATP + LZD (ATLZ).

### 2.5. Experimental Procedure

Liv-52 (50 mg/kg) [20] was administered orally by gavage to the LVLZ group (n = 6). The ATLZ group (n = 6) received ATP at a dose of 5 mg/kg via intraperitoneal (ip) injection [21]. The HG (n = 6) and LZDG (n = 6) groups were given saline (0.9% NaCl) as a solvent via the same route. One hour after the administration of Liv-52, ATP, and the solvent, linezolid (125 mg/kg) [22] was administered orally by the same method to the LZDG, LVLZ, and ATLZ groups. Liv-52 and ATP were administered once daily, while the linezolid treatment was repeated twice daily for 14 days. At the end of this period, the rats were sacrificed under high-dose anesthesia (thiopental sodium, 50 mg/kg, intraperitoneally), and liver tissues were collected. MDA, tGSH, SOD, and CAT levels were measured in the extracted liver tissues. In addition, NF-κB, MLKL, NLRP3, and RIPK3 immunopositivity were investigated using the double immunofluorescence method.

### 2.6. Biochemical Analyses

#### 2.6.1. Preparation of Samples

Liver tissues were carefully excised and rinsed with ice-cold 0.9% sodium chloride solution to remove residual blood and debris. Approximately 200 mg of tissue was weighed, finely minced, snap-frozen in liquid nitrogen, and pulverized using a pre-chilled mortar and pestle. The powdered tissue was homogenized in phosphate-buffered saline (PBS, pH 7.4) supplemented with protease inhibitors at a 1:10 (*w*/*v*) ratio using a mechanical homogenizer, with all procedures performed on ice. The homogenates were vortexed briefly and centrifuged at 10,000× *g* for 15 min at 4 °C. The resulting supernatants were collected and stored at −80 °C until analysis. Biochemical parameters were measured using commercially available assay kits according to the manufacturers’ instructions and normalized to total protein content. Results were expressed as nmol/mg protein for MDA and tGSH, and U/mg protein for SOD and CAT. Variations in absolute values were attributed to assay sensitivity and homogenization efficiency; therefore, statistical analyses focused on relative differences among groups.

#### 2.6.2. Quantification of MDA, tGSH, SOD and CAT Levels in Liver Tissue

Liver tissues were homogenized in ice-cold PBS and centrifuged at 10,000 rpm for 15 min at 4 °C. The supernatants were collected for biochemical analyses. MDA and tGSH levels, as well as SOD activity, were determined using commercially available colorimetric assay kits (catalog numbers: 10009055 for MDA, 703002 for tGSH, and 706002 for SOD; Cayman Chemical Co., Ann Arbor, MI, USA) according to the manufacturer’s instructions. Catalase activity was measured spectrophotometrically using Goth’s method [23]. Absorbance values were measured using a microplate reader, and the results were expressed per gram protein.

### 2.7. Histopathological Procedures

Liver tissues were fixed in 10% neutral buffered formalin, processed routinely, and embedded in paraffin. Sections of 5 μm thickness were obtained from paraffin blocks, deparaffinized in xylene, and rehydrated through a graded alcohol series (100%, 96%, 80%, and 70%). The sections were stained with hematoxylin and eosin (H&E) and examined under a light microscope. Histopathological alterations, including hepatocellular degeneration, necrosis, inflammatory cell infiltration, and vascular congestion, were evaluated by a blinded pathologist. The severity of these changes was scored semi-quantitatively as follows: absent (0), mild (1), moderate (2), and severe (3) [24].

### 2.8. Double Immunofluorescence Method

Paraffin-embedded tissue sections (5 μm) mounted on poly-L-lysine-coated slides were deparaffinized in xylene and rehydrated through a graded alcohol series, followed by washing with PBS. Antigen retrieval was performed using citrate buffer (pH 7.4) in a microwave oven (800 W for 2 × 5 min). After cooling, the sections were washed twice with PBS for 10 min each and incubated in PBS containing 0.25% Triton X-100 and gelatin for 10 min to enhance permeability. Subsequently, the sections were blocked with 5% bovine serum albumin (BSA) for 1 h at room temperature. After blocking, the sections were incubated overnight at 4 °C with the following primary antibody pairs diluted 1:200: monoclonal NF-κB (Elabscience, Houston, TX, USA, Cat. no. E-AB-22016) with polyclonal MLKL (BT-Lab, Shanghai, China, Cat. no. BT-AP11379), and monoclonal NLRP3 (Santa Cruz Biotechnology, Dallas, TX, USA, Cat. no. BF-8029) with polyclonal RIPK3 (BT-Lab, Cat. no. BT-AP12295). Following PBS washes, the sections were incubated for 45 min with a secondary antibody cocktail containing goat anti-mouse FITC (Jackson ImmunoResearch, West Grove, PA, USA, Cat. no. 115-095-003) and anti-rabbit Alexa Fluor 594 (Cell Signaling Technology, Danvers, MA, USA, Cat. no. 8889S), each diluted 1:100 in 1% BSA. After washing with PBS, the sections were treated with 10 mM CuSO_4_ and 50 mM NH_4_Cl solution for 10 min to reduce autofluorescence. The sections were then rinsed with distilled water and counterstained with DAPI (4′,6-diamidino-2-phenylindole). Fluorescence images were obtained using a Zeiss Axiolab 5 fluorescence microscope (Oberkochen, Germany) equipped with an Axiocam 305 color camera (Oberkochen, Germany) and Colibri 3 illumination system (Oberkochen, Germany). MLKL and RIPK3 immunopositivity were visualized in red, while NF-κB and NLRP3 immunopositivity were visualized in green. The staining intensity was evaluated semi-quantitatively using ZEN Blue 3.1 software and scored as absent (0), mild (1), moderate (2), and severe (3).

### 2.9. Statistical Analysis

All statistical analyses were performed using IBM SPSS Statistics software (Version 22.0, IBM Corp., Armonk, NY, USA). Data were expressed as mean ± standard deviation (SD). The normality of data distribution was evaluated using the Shapiro–Wilk test, and homogeneity of variances was assessed by Levene’s test. For biochemical parameters (MDA, tGSH, SOD, and CAT), intergroup comparisons were performed using one-way analysis of variance (One-way ANOVA). When a statistically significant difference was detected, post hoc multiple comparison analysis was conducted using Tukey’s test to determine differences between individual groups. Histopathological findings were evaluated using semi-quantitative scoring. Since histopathological scores represent ordinal data, intergroup comparisons were analyzed using the Kruskal–Wallis test. When significant differences were identified, pairwise comparisons were performed using the Dunn–Bonferroni post hoc test. Immunohistochemical staining intensities were assessed semi-quantitatively based on staining severity and distribution. The resulting scores were statistically analyzed using the Kruskal–Wallis test followed by Dunn’s multiple comparison test for intergroup differences. A *p*-value of <0.05 was considered statistically significant for all analyses.

## 3. Results

### 3.1. Biochemical Results

#### Tissue MDA, tGSH, SOD and CAT Levels

**Figure 1:**
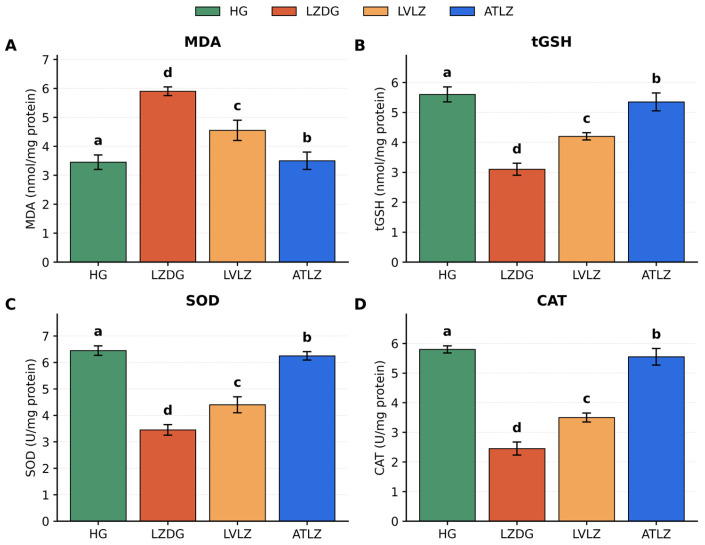
Effects of ATP and Liv-52 treatments on oxidative stress and antioxidant parameters in liver tissue. (**A**) MDA levels, (**B**) tGSH levels, (**C**) SOD activity, (**D**) CAT activity. Data are presented as mean ± SD. Statistical differences among groups were analyzed using one-way ANOVA followed by Tukey’s post hoc test. Different letters above the bars indicate statistically significant differences between groups (*p* < 0.05). HG: healthy group; LZDG: Linezolid group; LVLZ: Liv-52 + Linezolid group; ATLZ: ATP + Linezolid group.

As shown in Table 1 and Figure 1A, MDA levels differed significantly among the groups (*p* < 0.001). MDA levels increased in the LZDG group, whereas Liv-52 and ATP treatments reduced this increase. Notably, in the ATLZ group, MDA levels were close to those observed in the HG group.

One-way ANOVA showed a significant difference in tGSH levels among the groups (*p* < 0.001). tGSH levels decreased in the LZDG group, whereas Liv-52 and ATP treatments increased these levels. tGSH levels in the ATLZ group were comparable to those of the HG group (Table 1 and Figure 1B).

As presented in Table 1 and Figure 1C, SOD levels differed significantly among the groups (*p* < 0.001). SOD activity decreased in the LZDG group, whereas this reduction was attenuated in the LVLZ and ATLZ groups. Notably, SOD levels in the ATLZ group were similar to those of the HG group.

One-way ANOVA analysis revealed significant differences in CAT levels among the groups (*p* < 0.001). CAT activity decreased in the LZDG group, whereas this reduction was attenuated in the LVLZ and ATLZ groups. Notably, CAT levels in the ATLZ group were close to those of the HG group (Table 1 and Figure 1D).

### 3.2. Histopathological Results

**Figure 2:**
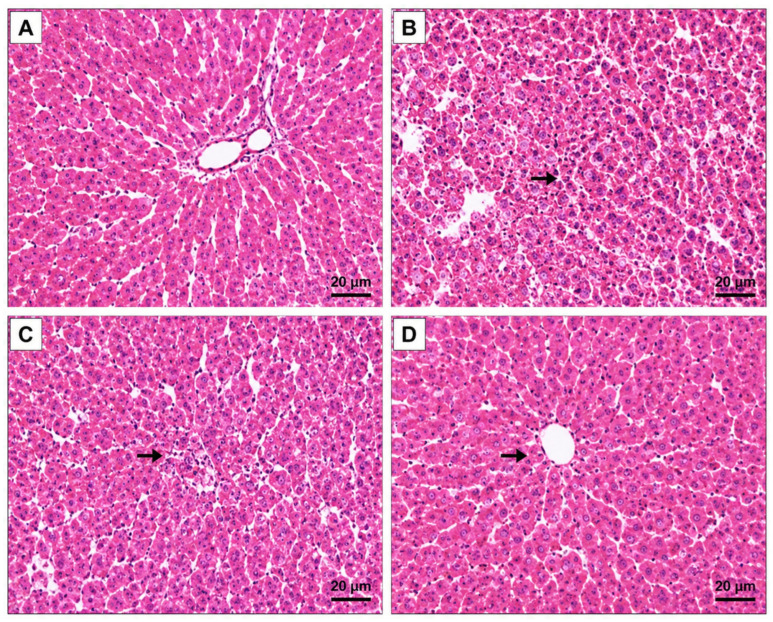
Representative histopathological findings in liver tissues in the experimental groups. (**A**) Normal histological architecture in the HG group. (**B**) Severe hepatocellular necrosis (→) in the LZDG group. (**C**) Moderate hepatocellular necrosis (→) in the LVLZ group. (**D**) Mild hepatocellular necrosis (→) in the ATLZ group. H&E staining, ×200 magnification. HG: Healthy group; LZDG: Linezolid group; LVLZ: Liv-52 + Linezolid group; ATLZ: ATP + Linezolid group.

Histopathological examinations revealed statistically significant differences among the experimental groups (Table 2). The HG group exhibited normal hepatic architecture together with preserved hepatocyte morphology (Figure 2A). In contrast, severe hepatocellular necrosis, cellular degeneration, and disrupted hepatic composition were predominant in the LZDG group (Figure 2B). In the LVLZ group, moderate hepatocellular necrosis and partially preserved tissue architecture were observed (Figure 2C). The ATLZ group demonstrated markedly reduced necrotic alterations compared to the LZDG group, and hepatocyte architecture was found to be largely preserved (Figure 2D).

### 3.3. Double Immunofluorescence Findings

**Figure 3:**
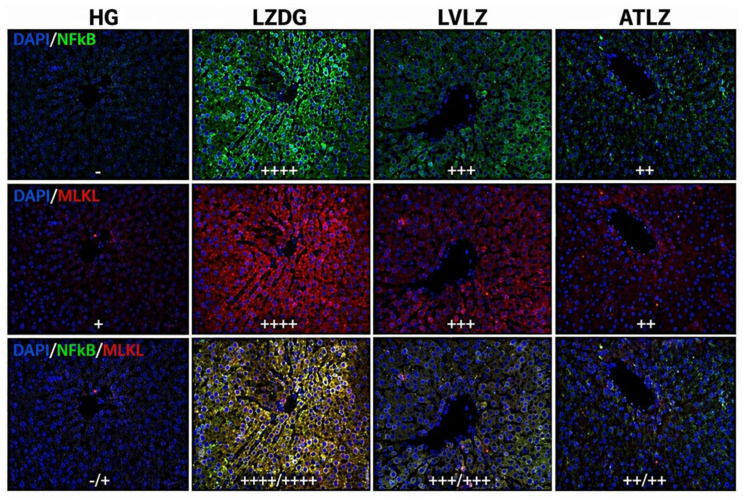
Fluorescence positivity of NF-κB and MLKL in HG, LZDG, LVLZ, and ATLZ groups. Immunostaining intensities were evaluated semi-quantitatively as negative (−), mild (+), moderate (++), severe (+++), and very severe (++++) positivity. NF-κB: FITC; MLKL: Alexa Fluor 595; DAPI: 4′,6-diamidino-2-phenylindole. HG: Healthy group; LZDG: Linezolid group; LVLZ: Liv-52 + Linezolid group; ATLZ: ATP + Linezolid group.

**Figure 4:**
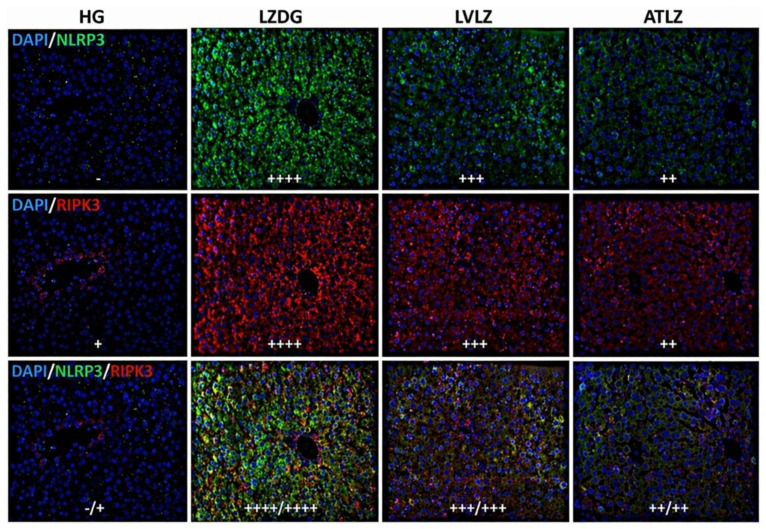
Fluorescence positivity of NLRP3 and RIPK3 in HG, LZDG, LVLZ, and ATLZ groups. Immunostaining intensities were evaluated semi-quantitatively as negative (−), mild (+), moderate (++), severe (+++), and very severe (++++) positivity. NLRP3: FITC; RIPK3: Alexa Fluor 595; DAPI: 4′,6-diamidino-2-phenylindole. HG: Healthy group; LZDG: Linezolid group; LVLZ: Liv-52 + Linezolid group; ATLZ: ATP + Linezolid group.

As shown in Table 3 and Figure 3, immunofluorescence staining for NF-κB in liver tissues revealed no detectable positivity in the HG group, whereas very strong, strong, and moderate immunopositivity were observed in the LZDG, LVLZ, and ATLZ groups, respectively. MLKL immunopositivity was detected at a mild level in the HG group, while it was very strong, strong, and moderate in the LZDG, LVLZ, and ATLZ groups, respectively. Furthermore, as demonstrated in Table 3 and Figure 4, NLRP3 immunopositivity was not observed in the HG group, whereas it was very strong in the LZDG group and strong and moderate in the LVLZ and ATLZ groups, respectively. Similarly, RIPK3 staining showed mild immunopositivity in the HG group, while very strong, strong, and moderate levels were observed in the LZDG, LVLZ, and ATLZ groups, respectively.

## 4. Discussion

In this study, the protective effects of ATP and Liv-52 against LZD-induced liver injury were investigated using biochemical, histopathological, and immunofluorescence methods. The obtained data demonstrated that prolonged LZD administration induced marked oxidative stress, as evidenced by increased levels of the oxidant parameter MDA and significant decreases in the antioxidant parameters tGSH, SOD, and CAT. These biochemical alterations were accompanied by pronounced histopathological damage and increased expression of inflammatory and apoptotic markers, as revealed by immunofluorescence analysis. Our findings indicate that LZD has a substantial hepatotoxic potential, and that ATP, compared to Liv-52, exerts a more pronounced protective effect by ameliorating this damage.

Disruption of oxidative phosphorylation, depletion of ATP levels, and increased production of ROS are among the principal mechanisms underlying liver injury [5]. ROS suppress endogenous antioxidant defense systems, leading to redox imbalance, enhanced oxidative stress, and inflammation [6,25]. MDA is one of the most widely used and reliable biomarkers of ROS-induced lipid peroxidation [26]. LZD, an antibiotic effective against Gram-positive bacteria, has been reported to induce mitochondrial dysfunction during prolonged use [1]. As a consequence, oxidative phosphorylation is impaired, cellular ATP levels decline, and lactic acidosis develops, ultimately resulting in organ toxicity [27]. The pathogenesis of LZD-induced oxidative stress has been attributed to increased levels of MDA, a byproduct of ROS, along with decreased levels of endogenous antioxidant biomarkers such as GSH, SOD, and CAT [28]. In the present study, MDA levels in the liver tissues of animals treated with LZD alone were significantly higher compared to those in the other groups, indicating that LZD promotes oxidative stress by enhancing lipid peroxidation. These findings are consistent with previous reports [1]. tGSH, SOD, and CAT constitute major antioxidant defense mechanisms that protect cells against oxidative stress-induced damage [29]. Numerous studies in the literature have demonstrated the tissue-protective effects of antioxidant agents against oxidative damage [7]. Vivekanandan et al. (2018) reported that silymarin prevents the LZD-induced decrease in hepatic antioxidant enzymes such as SOD, CAT, and GPx, and attenuates the increase in MDA levels, thereby restoring antioxidant balance by suppressing oxidative damage [2]. It has also been reported that LZD increases oxidative stress while reducing antioxidant defenses (GSH and SOD), leading to enhanced lipid peroxidation in the liver, as evidenced by elevated MDA levels [30]. Consistent with these findings, our results demonstrated that the levels of tGSH, SOD, and CAT—key components of the antioxidant defense system—were significantly decreased in the group treated with LZD alone. These findings suggest that LZD induces a comprehensive pattern of hepatic injury by suppressing cellular antioxidant defense mechanisms at the mitochondrial level.

Liv-52 is a compound with antioxidant, anti-inflammatory, and immunomodulatory properties [15,16]. Its protective mechanism is attributed to the prevention of GSH depletion and a reduction in LPO [17]. This suggests that Liv-52 may be beneficial in modulating drug-induced oxidative stress in the liver. In the literature, Liv-52 administration has been shown to prevent drug-induced increases in MDA levels [16] while attenuating decreases in tGSH and SOD activities [31]. Vidyashankar and Patki (2010) reported that reductions in SOD, CAT, and tGSH levels associated with hepatic toxicity were inhibited by Liv-52 [32]. Consistent with previous reports, Liv-52 in our study inhibited the increase in MDA levels and prevented the decrease in tGSH, SOD, and CAT levels induced by LZD in liver tissue.

Although oxidative stress and inflammation are recognized as key mechanisms underlying LZD-induced toxicity, previous studies have also reported that the drug reduces intracellular ATP concentrations [9]. Moreover, extracellular ATP exerts its effects primarily through purinergic receptors, which play a role in the regulation of oxidative stress and inflammation [33]. In liver injury, disruption of mitochondrial respiratory chain (MRC) coupling leads to hepatic ATP depletion [34]. Decreased ATP levels, together with increased ROS production, initiate oxidative stress-mediated processes, including cell death, inflammation, and fibrosis [35]. Our findings demonstrated that there was no significant difference between the HG and ATLZ groups in terms of oxidant and antioxidant levels in liver tissues. The increase in oxidant parameters and the decrease in antioxidant molecules observed in LZD-treated animals were effectively suppressed by ATP. Consistent with the literature, exogenous ATP administration has been shown to prevent increases in MDA levels and decreases in antioxidant defense enzymes, including tGSH, SOD, and CAT, in drug-induced hepatotoxicity [36]. Karadoğan et al. (2025) further demonstrated that ATP exerts hepatoprotective effects by reducing oxidative stress and enhancing antioxidant defense mechanisms in drug-induced liver injury [37].

The detection of significant differences among the groups in histopathological evaluation demonstrated that linezolid induces marked structural damage in liver tissue, supporting our biochemical findings. The presence of severe hepatocellular necrosis in the group treated with LZD alone is consistent with previous reports indicating that the drug causes hepatocellular injury through mitochondrial dysfunction and oxidative stress [38]. Indeed, in models of drug-induced hepatotoxicity, mitochondrial impairment, ATP depletion, and increased production of reactive oxygen species have been reported to result in hepatocyte necrosis [39]. The observation of moderate necrosis in the LVLZ group suggests that Liv-52 exerts a partial protective effect by enhancing antioxidant defense mechanisms in liver tissue. Previous histopathological studies have shown that Liv-52 reduces lipid peroxidation and alleviates hepatic damage by enhancing antioxidant enzyme activities [20]. In contrast, the marked attenuation of hepatocellular necrosis in the ATLZ group indicates that ATP may limit cellular injury by supporting mitochondrial energy metabolism and reducing oxidative stress. Experimental hepatotoxicity models have reported that exogenous ATP preserves mitochondrial function, prevents ATP depletion, and consequently reduces histopathological liver damage [16,40].

The potential protective effects of Liv-52 and ATP on LZD-induced liver injury, as well as their relationship with inflammatory and necroptotic pathways, were evaluated in our study using immunofluorescence analysis. The absence of NF-κB immunopositivity in the healthy group indicates that the inflammatory response is suppressed under physiological conditions. In contrast, the very strong positivity observed in the LZD group is consistent with previous reports suggesting that the drug induces oxidative stress by inhibiting mitochondrial protein synthesis and subsequently activates inflammatory pathways [4,27]. Increased oxidative stress enhances the expression of proinflammatory cytokines such as TNF-α, IL-1β, and IL-6 via NF-κB activation [41]. It has also been reported that LZD administration increases the expression of immunofluorescence markers associated with inflammation and cell death, particularly NF-κB, in liver tissue [1]. The reduction in these expression levels in the antioxidant-treated groups demonstrates that LZD-induced damage is mediated by oxidative stress. The decreased immunopositivity observed in the ATLZ and LVLZ groups further supports the anti-inflammatory effects of these agents [40]. Evaluation of the immunofluorescence findings revealed that ATP exerted a more pronounced protective effect compared to Liv-52. The marked increase in MLKL and RIPK3 expression indicates that LZD activates necroptotic cell death pathways. Mitochondrial damage and oxidative stress are known to trigger necroptosis [42]. The reduction in these expression levels following antioxidant treatment supports previous reports demonstrating that Liv-52 decreases markers associated with inflammation and cell death in experimental hepatotoxicity models [14,41]. The significant increase in NLRP3 inflammasome expression in the LZD group is consistent with oxidative stress-mediated inflammasome activation, as ROS have been identified as key triggers of NLRP3 activation in the literature [43]. The decreases observed in the ATP- and Liv-52-treated groups indicate that these agents suppress inflammasome-mediated inflammatory responses. The antioxidant and hepatoprotective effects of Liv-52 are well established in the literature [15,16]. Likewise, ATP is one of the most important agents contributing to the maintenance of mitochondrial functions [42].

This study has several limitations. First, the use of a rat experimental model limits the direct translational applicability of the findings to clinical settings. Although immunofluorescence evaluations provided important insights into inflammatory and necroptotic pathways, the absence of molecular validation techniques such as Western blot or qPCR represents an important limitation. In addition, tissue ATP levels were not directly measured in linezolid-treated animals, limiting the ability to clearly determine alterations in endogenous ATP content and limiting the direct evaluation of the proposed ATP replenishment mechanism. Oxidative stress was evaluated indirectly using biochemical markers, whereas direct ROS measurements and mitochondrial functional analyses, including mitochondrial membrane potential and respiratory chain activity, were not performed. Furthermore, the use of a single dose and the absence of long-term administration protocols for ATP and Liv-52 restricted the evaluation of dose–response relationships and chronic exposure effects. Another limitation of the present study is the absence of ATP-only and Liv-52-only control groups, which restricts the evaluation of the independent effects of these agents on normal liver function. Additionally, exogenous ATP is inherently unstable and may be rapidly degraded by ectonucleotidases such as CD39 and CD73, potentially limiting its bioavailability. The purinergic receptor-mediated effects of ATP at the cellular level should also be considered. Future studies involving advanced molecular analyses, mitochondrial functional assessments, tissue ATP quantification, and different dose regimens are needed to further clarify the underlying protective mechanisms.

## 5. Conclusions

In conclusion, the present study demonstrated that linezolid induces significant hepatotoxicity through oxidative stress, inflammatory responses, and activation of necroptotic pathways in liver tissue. Increased NF-κB, NLRP3, RIPK3, and MLKL expression levels indicated that both inflammatory and necroptotic mechanisms play critical roles in LZD-induced liver injury. Treatment with ATP and Liv-52 effectively ameliorated the biochemical, histopathological, and molecular alterations associated with hepatotoxicity. Notably, ATP exhibited more pronounced hepatoprotective effects than Liv-52 through the regulation of mitochondrial functions and oxidative stress. These findings suggest that ATP may represent a promising therapeutic approach for the prevention of drug-induced liver injury. In addition, ATP and Liv-52 may have potential as adjunctive therapeutic agents against linezolid-induced hepatotoxicity; however, extensive further studies regarding safety, efficacy, optimal dosing, and pharmacokinetic properties are required before clinical application can be considered. Further extensive experimental and clinical studies are needed to better elucidate the underlying molecular mechanisms and evaluate the potential clinical applicability of ATP in hepatotoxic conditions.

## Figures and Tables

**Table 1:** Effects of ATP and Liv-52 on oxidative stress and antioxidant parameters in liver tissue.

Groups	MDA (nmol/mg Protein)	tGSH (nmol/mg Protein)	SOD (U/mg Protein)	CAT (U/mg Protein)
HG	3.42 ± 0.27 ^a^	5.58 ± 0.23 ^a^	6.39 ± 0.21 ^a^	5.76 ± 0.13 ^a^
LZDG	5.87 ± 0.15 ^d^	3.12 ± 0.18 ^d^	3.46 ± 0.24 ^d^	2.45 ± 0.22 ^d^
LVLZ	4.57 ± 0.36 ^c^	4.21 ± 0.13 ^c^	4.40 ± 0.28 ^c^	3.50 ± 0.13 ^c^
ATLZ	3.55 ± 0.36 ^b^	5.34 ± 0.27 ^b^	6.22 ± 0.16 ^b^	5.55 ± 0.25 ^b^

Data are presented as mean ± SD. Statistical differences among groups were analyzed using one-way ANOVA followed by Tukey’s post hoc test. Different superscript letters indicate significant differences between groups (*p* < 0.05). HG: Healthy group; LZDG: Linezolid group; LVLZ: Liv-52 + Linezolid group; ATLZ: ATP + Linezolid group.

**Table 2:** Histopathological evaluation of hepatocyte necrosis in experimental groups.

Groups	Hepatocellular Necrosis (Score, Median [Min–Max])
HG	0 (0–0) ^a^
LZDG	3 (3–4) ^d^
LVLZ	3 (2–3) ^c^
ATLZ	1 (1–2) ^b^

Data are expressed as median (min–max). Histopathological scores were analyzed using the Kruskal–Wallis test followed by Dunn–Bonferroni post hoc multiple comparison analysis. Different superscript letters (a–d) indicate statistically significant differences between groups (*p* < 0.05). HG: Healthy group; LZDG: Linezolid group; LVLZ: Liv-52 + Linezolid group; ATLZ: ATP + Linezolid group.

**Table 3:** Double immunofluorescence analysis of NF-κB, MLKL, NLRP3, and RIPK3 in liver tissues of experimental groups.

Groups	NF-κB	MLKL	NLRP3	RIPK3
HG	0 (0–0) ^a^	1 (0–1) ^a^	0 (0–0) ^a^	1 (0–1) ^a^
LZDG	4 (3–4) ^d^	4 (3–4) ^d^	4 (3–4) ^d^	4 (3–4) ^d^
LVLZ	3 (2–3) ^c^	3 (2–3) ^c^	3 (2–3) ^c^	3 (2–3) ^c^
ATLZ	2 (1–2) ^b^	2 (1–2) ^b^	2 (1–2) ^b^	2 (1–2) ^b^

Data are expressed as median (min–max). Immunostaining intensities were evaluated semi-quantitatively. Statistical comparisons were performed using the Kruskal–Wallis test followed by Dunn–Bonferroni post hoc analysis. Different superscript letters (a–d) indicate statistically significant differences between groups (*p* < 0.05). HG: Healthy group; LZDG: Linezolid group; LVLZ: Liv-52 + Linezolid group; ATLZ: ATP + Linezolid group.

## Data Availability

The datasets generated and/or analyzed during the current study are available from the corresponding author on request.

## Abbreviations

The following abbreviations are used in this manuscript:

ATP	Adenosine triphosphate
MDA	Malondialdehyde
tGSH	Total glutathione
SOD	Superoxide dismutase
CAT	Catalase
ROS	Reactive oxygen species
LPO	Lipid peroxidation
ELISA	Enzyme-Linked Immunosorbent Assay
H&E	Hematoxylin and eosin
PBS	Phosphate-buffered saline
